# Stress induces insertion of calcium-permeable AMPA receptors in the OFC–BLA synapse and modulates emotional behaviours in mice

**DOI:** 10.1038/s41398-020-0837-3

**Published:** 2020-05-18

**Authors:** Hiroshi Kuniishi, Daisuke Yamada, Keiji Wada, Mitsuhiko Yamada, Masayuki Sekiguchi

**Affiliations:** 1 Department of Degenerative Neurological Diseases, National Institute of Neuroscience, National Center of Neurology and Psychiatry, Kodaira, Tokyo Japan; 2 Department of Neuropsychopharmacology, National Institute of Mental Health, National Center of Neurology and Psychiatry, Kodaira, Tokyo Japan

**Keywords:** Molecular neuroscience, Physiology

## Abstract

Stress increases the risk of neuropsychiatric disorders, such as major depression. Exposure to stress has been reported to induce various neuronal changes, such as alterations in synaptic transmission and structure. However, a causal link between stress-induced neural circuit alterations and changes in emotional behaviours is not well understood. In the present study, we focused on a projection pathway from the orbitofrontal cortex (OFC) to the basolateral nucleus of the amygdala (BLA) as a crucial circuit for negative emotions and examined the effect of stress on OFC–BLA excitatory synaptic transmission using optogenetic and whole-cell patch-clamp methods in mice. As a stress-inducing procedure, we used repeated tail-shock, which increased stress-related behaviours. We found greater α-amino-3-hydroxy-5-methyl-4-isoxazole propionic acid (AMPA)/N-methyl-d-aspartate current ratios and insertion of calcium-permeable AMPA receptors (AMPARs) in the OFC–BLA synapse after stress. These stress-induced synaptic and behavioural changes were reduced by a blockade of protein kinase A, which plays a principal role in stress-induced targeting of AMPARs into the synaptic membrane. To examine a possible causal relationship between alterations in synaptic transmission in the OFC–BLA pathway and stress-related behaviour, we performed optogenetic activation or chemogenetic inactivation of OFC–BLA transmission in mice. We found that optogenetic activation and chemogenetic inactivation of OFC–BLA transmission increased and decreased stress-related behaviour, respectively. In conclusion, we have demonstrated that stress altered the postsynaptic properties of the OFC–BLA pathway. These synaptic changes might be one of the underlying mechanisms of stress-induced behavioural alterations.

## Introduction

The orbitofrontal cortex (OFC), a ventral subregion of the prefrontal cortex (PFC), has recently been highlighted as a critical region in stress-related psychiatric disorders such as depression^1^. Recent studies have shown that greater activity is observed in the OFC in depressed patients and stressed animals^2,3^. Pharmacological inactivation of the OFC has been reported to decrease stress-related behaviour in rats^4,5^. These findings suggest that the OFC is involved in stress-related psychiatric symptoms in patients, and behavioural abnormalities in stressed animals. The OFC shares reciprocal connections with brain structures implicated in emotional processing^6–9^. Among these, the amygdala receives dense projections from the OFC^6,7^ and has been proposed to be one of the key structures in the generation of negative emotions^10,11^. Similar to the OFC, hyperactivation of the amygdala has also been observed in patients with stress-related psychiatric disorders, and in stressed experimental animals^2,12,13^. Moreover, the functional connectivity between the OFC and the amygdala has been found to be enhanced in depressive patients^14–16^. Taken together, it is proposed that stress affects OFC–amygdala synaptic transmission, which underlies stress-induced behavioural abnormalities and stress-related psychiatric symptoms.

The α-amino-3-hydroxy-5-methyl-4-isoxazole propionic acid receptors (AMPARs) are the primary mediators of fast excitatory transmission. Interestingly, altered expression, phosphorylation, and subcellular localisation of AMPARs have been observed in several limbic regions in stressed animals^17–22^. Stress activates the hypothalamus–pituitary–adrenal axis and results in the release of glucocorticoids (GCs). Recent studies have reported that GCs could modulate excitatory transmission through the regulation of AMPAR trafficking^20,23^. These findings suggest that stress disrupts emotional information processing through a change in AMPAR-mediated excitatory transmission, which underlies stress-induced behavioural abnormalities. However, a causal relationship between stress-induced changes in AMPAR-mediated synaptic responses and stress-related behaviours has not been adequate.

Therefore, to investigate the effect of stress on excitatory transmission in the OFC–amygdala pathway, we first isolated OFC–amygdala excitatory synaptic transmission using optogenetic methods in mice. Then, we examined the effects of repeated tail-shock stress on excitatory transmission using whole-cell patch-clamp methods. To clarify the relationship between OFC–amygdala excitatory transmission and stress-related behaviour, we examined the effect of optogenetic activation and chemogenetic inactivation of OFC–amygdala transmission on stress-related behaviours in mice.

## Material and methods

Detailed procedures are described in Supplementary Information.

### Animals and stress procedure

Male C57BL/6J mice (3–5 weeks of age at viral injection surgery, 8–12 weeks of age for electrophysiological experiments and behavioural tests, CLEA, Tokyo, Japan) were used. The experimental procedures met the standards established in the guidelines of the National Institute of Neuroscience, and National Center of Neurology and Psychiatry, and were approved by the Institutional Animal Investigation Committee.

As a stress procedure, mice received repeated tail-shock stress^24^ for 3 consecutive days. Mice were subjected to behavioural or electrophysiological tests within 2 days after their last stress session.

### Behavioural tests

To evaluate stress-related behaviour, we conducted the forced swim test (FST, 6 min), and the tail-suspension test (TST, 6 min). To evaluate locomotor activity, we conducted the open field test (OFT, 6 or 5 min).

### Stereotaxic surgery

Animals were anaesthetised with an intraperitoneal injection of ketamine (100 mg/kg) and xylazine (20 mg/kg). An adeno-associated virus (AAV) vector (AAV5-CaMKIIa-ChR2-(H134R)-EYFP, and AAV5-CaMKIIa-EYFP were purchased from the University of North Carolina Vector Core, and AAV5-CaMKIIa-hM4Di-mCherry was purchased from Addgene) was infused into the OFC or anterior cingulate cortex (ACC) using a stereotaxic instrument (Narishige, Tokyo, Japan). The mice had a guide cannula (Eicom, Kyoto, Japan) implanted into their lateral ventricle or the basolateral nucleus of their amygdala (BLA) for drug microinjection. The mice had a dual-LED optic cannula (TeleLCD-B-5-500-6.2, BRC Nihon Bioresearch, Hashima, Japan) implanted into their BLA 3–4 weeks after AAV injection for optogenetic activation.

### Electrophysiology

Whole-cell patch-clamp recording from pyramidal neurons in the amygdala was performed as described previously^25–28^ (see Supplementary Information). Patch electrodes were filled with a solution containing 100 μM spermine when the voltage–current relationship for AMPA currents was examined.

### Drug microinjection

Before drug infusion, mice were gently restrained, and dummy cannula were replaced with injection cannula that extended 1 mm from the tip of the guide cannula. For protein kinase A (PKA) inhibition during a stress-inducing session, saline or Rp-cAMP (Santa Cruz Biotechnology, TX, USA) was infused into the lateral ventricle or the BLA using a 10 μL Hamilton syringe under infusion pump control. For chemogenetic inhibition of OFC–BLA transmission, saline or clozapine n-oxide (CNO, Hello Bio, Bristol, UK) were infused into the BLA.

### Optogenetic stimulation

Optogenetic stimulation was performed using a wireless optogenetic stimulation system (Teleopto, BRC Nihon Bioresearch).

### Statistical analysis

Sample sizes were based on previous studies that undertook similar behavioural analyses^29,30^. Values are expressed as a mean ± standard error of the mean. All statistical analyses were performed using EZR statistical software^31^. All data were analysed using the Shapiro–Wilk test to examine sample distribution and analysed using the *F*-test to examine homoscedasticity. Statistical comparisons between two groups were carried out using two-sided unpaired *t*-tests for Gaussian distribution, or the Mann–Whitney *U*-test for non-Gaussian distribution. For the unpaired *t*-tests, homoscedastic and heteroscedastic data were analysed by the Student’s *t*-test and Welch’s *t*-test, respectively. For multiple comparisons, data were analysed using a two-way ANOVA followed by the Tukey’s Honest Significant Difference (HSD) post hoc test. The effect of drugs on the amplitude of the excitatory postsynaptic current (EPSC) was analysed using a two-way ANOVA followed by an unpaired *t*-test. A *P*-value of < 0.05 was considered statistically significant.

## Results

### Optogenetic isolation of OFC–amygdala synaptic transmission

**Fig. 1:**
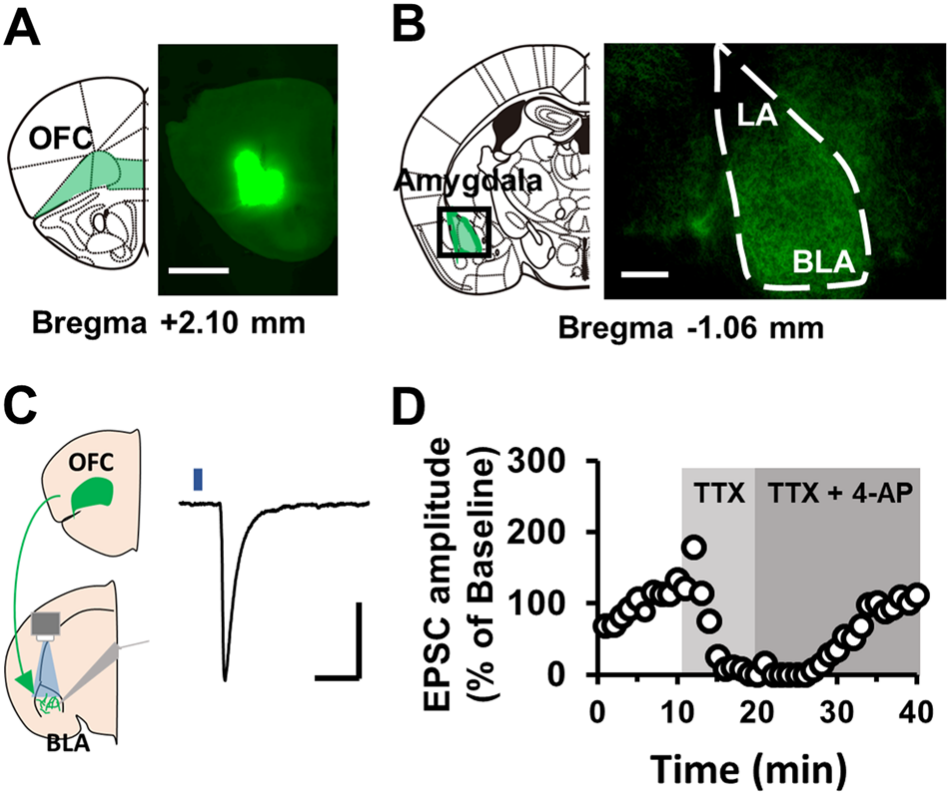
Optogenetic isolation of OFC–BLA synaptic transmission. **a**, **b** Representative photographs of ChR2-EYFP fluorescence at the viral OFC injection site (**a**, scale bar: 1000 μm) and ChR2-EYFP expressing afferent OFC axon in the BLA (**b**, scale bar: 250 μm). **c** Schematic of optogenetic isolation of the OFC–BLA synaptic response (left) and a typical light-evoked excitatory postsynaptic current (EPSC) recorded from a BLA pyramidal neuron in response to blue light irradiation from a ×63 objective lens (blue square), during whole-cell patch-clamp recording at −70 mV (right). Scale: 20 ms and 50 pA. **d** The light-evoked EPSC was abolished by perfusion with tetrodotoxin (TTX) and recovered after additional perfusion with 4-aminopyridine (4-AP).

To isolate OFC–amygdala synaptic transmission, we injected an AAV vector expressing a ChR2-EYFP fusion protein under the control of a CaMKIIα promoter into the lateral part of the OFC in mice. Four weeks after injection, ChR2-EYFP was expressed in the lateral and ventral regions of the OFC without spread to the medial region of the OFC (Fig. 1a) and, ChR2-EYFP expressing axon terminals were observed in the anterior part of the BLA, consistent with previous studies^32,33^ (Fig. 1b). We then conducted whole-cell recordings in BLA pyramidal neurons in acute brain slices obtained from the mice. In the BLA, ChR2 expressing axons were activated by local blue light irradiation, and light-evoked synaptic transmissions were recorded (Fig. 1c). Consistent with our previous studies on another synapse type^26^, the light-evoked-synaptic responses were completely suppressed by perfusion with tetrodotoxin (1 μM), and recovered by further perfusion with 4-aminopyridine (1 mM, Fig. 1d), indicating that they were ChR2-evoked monosynaptic responses^34^.

### Repeated tail-shock induced stress-related behavioural changes and altered postsynaptic plasticity in OFC–BLA excitatory transmission

**Fig. 2:**
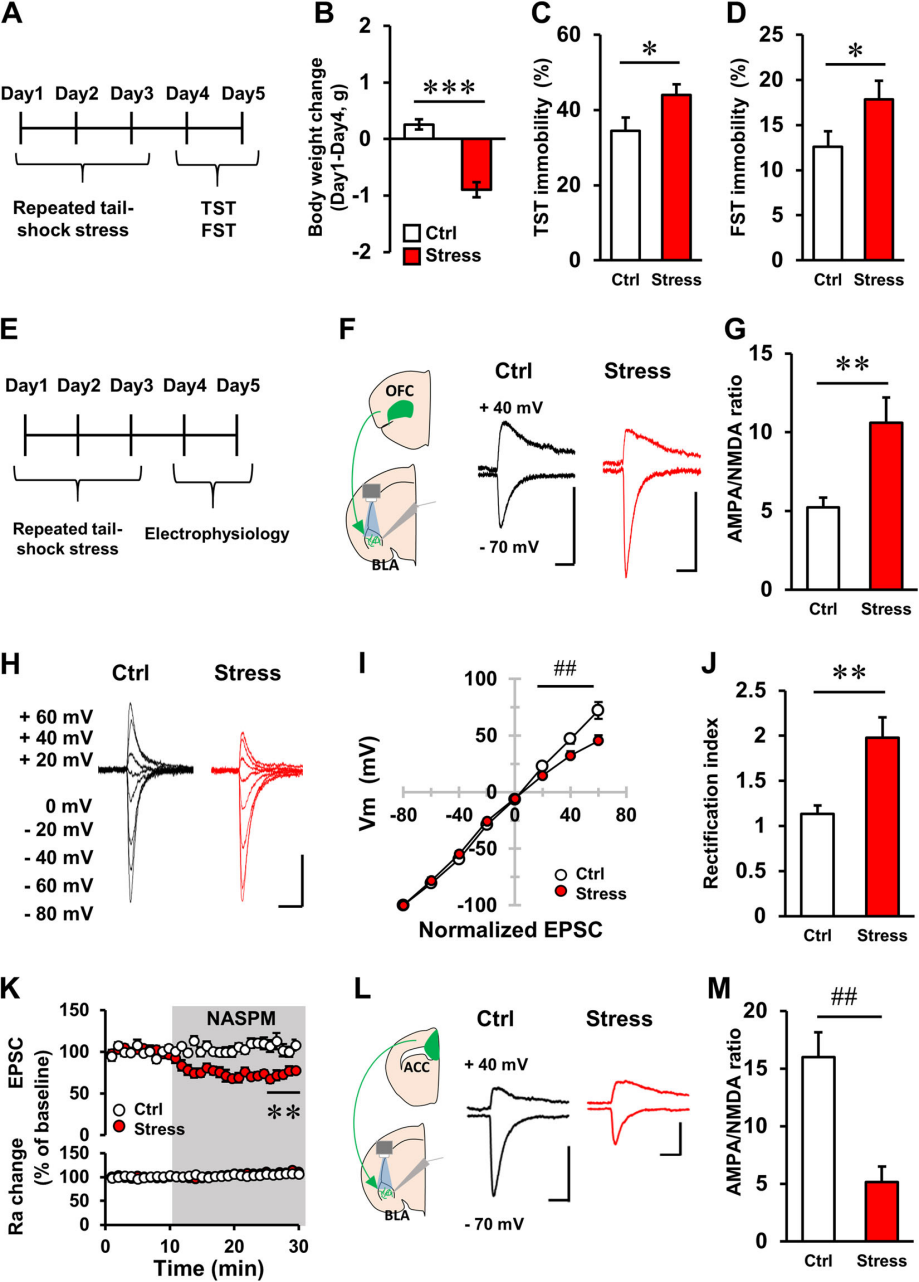
Repetitive tail-shock stress increased the AMPA/NMDA ratio and inward-rectification of AMPAR currents in the OFC–BLA synapse. **a** Time schedule of behavioural experiments. **b** Bodyweight loss induced by repetitive tail-shock stress induction. Mice were weighed on days 1 and 4. Ctrl: control animals, stress: stressed animals. **c**, **d** The effect of repetitive tail-shock on immobility in the tail suspension test (TST, **c**, Ctrl: *n* = 15, stress: *n* = 16) and the forced swim test (FST, **d**, Ctrl: *n* = 12, stress: *n* = 13). **e** Time schedule of physiological experiments. **f** Representative traces from AMPAR and AMPAR/NMDAR mixed EPSCs in the OFC–BLA synapse obtained from Ctrl (left) and stress animals (right). Scale: 20 ms and 100 pA. **g** The effects of repetitive tail-shock on the AMPA/NMDA ratio in the OFC–BLA synapse. Ctrl: *n* = 12 cells from 5 mice, stress: *n* = 12 cells from 4 mice. **h** Representative traces of AMPAR current at various holding potentials in OFC–BLA synapses of Ctrl (left) and stressed animals (right). Scale: 20 ms and 100 pA. **i** The effects of repetitive tail-shock on the current–voltage relationship of AMPAR-mediated current in the OFC–BLA synapse. Ctrl: *n* = 11 cells from 5 mice, stress: *n* = 12 cells from 4 mice. **j** The effect of repetitive tail-shock on the rectification index of AMPAR-mediated current (EPSC at −60 mV/EPSC at +60 mV) in the OFC–BLA synapse. Ctrl: *n* = 11 cells from 5 mice, stress: *n* = 12 cells from 4 mice. **k** The effect of NASPM on AMPAR EPSCs in the OFC–BLA synapse (top) and input resistance (bottom). Ctrl: *n* = 12 cells from 7 mice, stress: *n* = 12 cells from 8 mice. **l** Representative traces of AMPAR and AMPAR/NMDAR mixed EPSCs in the ACC–BLA synapse obtained from Ctrl (left) and stressed animals (right). **m** The effects of repetitive tail-shock on the AMPA/NMDA ratio in the ACC–BLA synapse. Ctrl: *n* = 11 cells from 4 mice, stress: *n* = 11 cells from 5 mice. *P < 0.05, **P < 0.01, ***P < 0.001, unpaired *t*-test. ^##^*P* < 0.01, Mann–Whitney *U*-test. Scale: 20 ms and 100 pA.

To induce stress in mice, we used a repeated tail-shock procedure, which induces several behavioural and physiological changes in rodents^24,35–37^ (Fig. 2a). After 3 consecutive days of stress-inducing sessions, stressed mice displayed significantly reduced body weights compared with the control mice (Fig. 2b, *t*(29) = 7.401, *P* < 0.001, Student’s *t*-test). To measure rodent stress-related behaviour, we performed tail-suspension (TST) and forced swim tests (FST). Previous studies have demonstrated that chronic stress and anti-depressant treatments increased and decreased immobility in these tests, respectively^22,30,38,39^. Consistent with previous reports, stressed mice displayed increased immobility in TST (Fig. 2c, *t*(29) = −2.101, *P* = 0.044, Student’s *t*-test) and FST (Fig. 2d, *t*(23) = −2.095, *P* = 0.047, Student’s *t*-test) compared with the control mice. These data indicate that repeated tail-shock stress is sufficient to induce behavioural abnormalities in mice.

Next, we examined the effect of repeated stress on postsynaptic plasticity in the OFC–BLA synapse. Four to seven weeks after AAV injection, mice received repeated-tail shock stress induction (Fig. 2e). First, we measured the relative ratio of AMPAR- to N-methyl-d-aspartate receptor (NMDAR)-mediated currents in excitatory transmission (AMPA/NMDA ratio), to provide a history of past plasticity in glutamatergic synapses. In stressed animals, the AMPA/NMDA ratio in OFC–BLA transmission was increased compared with the control animals (Fig. 2f, g, *t*(16.583) = −3.216, *P* = 0.004, Welch’s *t*-test). In the amygdala, synaptic strengthening is associated with the synaptic insertion of inwardly rectifying calcium-permeable AMPARs^40^ (CP-AMPARs). To examine whether CP-AMPARs were recruited into the OFC–BLA synapse by stress, we compared the current–voltage relationships of AMPARs in OFC–BLA synapses between control and stressed animals. Stressed mice showed greater inward rectification than control animals (Fig. 2h, i, at +20 mV, +40 mV, and +60 mV, *P* < 0.01, Mann–Whitney *U*-test, Fig. 2j, *t*(14.658) = −3.4333, *P* = 0.004, Welch’s *t*-test). In stressed mice, sensitivity to the CP-AMPAR blocker, NASPM was increased compared with control mice (Fig. 2k, 26–30 min, the main effect of stress: *P* < 0.001, two-way ANOVA. *P* < 0.01, Student’s *t*-test). These data suggest that repeated tail-shock induced recruitment of AMPARs, including CP-AMPARs in the OFC–BLA synapse.

### Repeated tail-shock stress-induced pathway-specific postsynaptic plasticity in two distinct prefrontal-amygdala pathways

We examined the effect of repeated tail-shock stress on another pathway connected to the BLA, the ACC–BLA synapse. To isolate ACC–BLA synaptic transmission, we injected an AAV vector coding ChR2-EYFP into the ACC. Four to seven weeks after injection, ChR2-EYFP expressing axon terminals were observed in the BLA^41,42^ (Supplementary Fig. 1A). Whole-cell recordings were performed in BLA pyramidal neurons in these animals, and light-evoked EPSCs (stimulated with blue light irradiation) were recorded (Fig. 2l). Interestingly, in contrast to the OFC–BLA synapse, stressed mice displayed decreased AMPA/NMDA ratios in ACC–BLA transmission compared with control mice (Fig. 2l, m, *U*(11,11) = 111, *P* < 0.001, Mann–Whitney *U*-test). To examine the effect of stress on nonspecific-synaptic input to pyramidal neurons in the BLA, we recorded the AMPA/NMDA ratio obtained by intra-BLA electrical stimulation (Supplementary Fig. 1B). We observed no significant change in the AMPA/NMDA ratios obtained by intra-BLA electrical stimulation (Supplementary Fig. 1D, E, *U*(11,11) = 72, *P* = 0.478, Mann–Whitney *U*-test). These data suggest that repeated tail-shock stress-induced pathway-specific postsynaptic plasticity in two distinct prefrontal-amygdala pathways.

To assess the effect of stress on the presynaptic short-term plasticity, we delivered paired-pulse blue light stimulation with various range of interstimulus intervals (50, 100, 250, 500, and 1000 ms) and calculated the paired-pulse ratio in the OFC–BLA and ACC–BLA synapses. There were no significant differences in the paired-pulse ratio between stressed mice and control mice in both the OFC–BLA and ACC–BLA synapses (Supplementary Fig. 2A–D, *P* > 0.05, Student’s *t*-test). These data suggest that repeated tail-shock stress did not significantly alter presynaptic release probability in these two pathways.

### PKA inhibition prevented stress-induced synaptic change in the OFC–BLA synapse, accompanied by a blockade of stress-induced behavioural changes

**Fig. 3:**
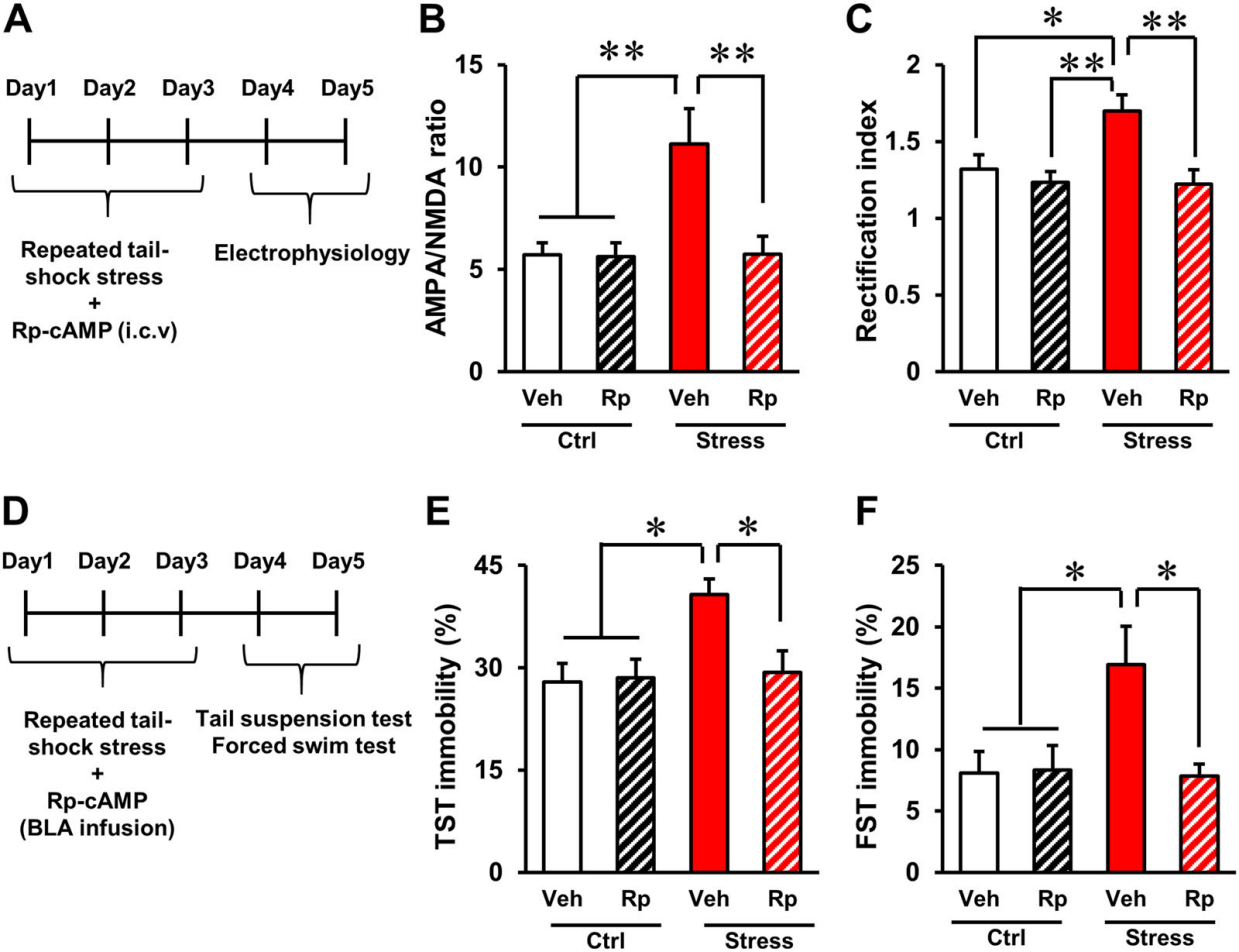
PKA inhibition prevented stress-induced postsynaptic changes in the OFC–BLA pathway and stress-induced behavioural changes. **a** Time schedule of experiments examining the effect of PKA inhibition on stress-induced synaptic changes in the OFC–BLA synapse. Mice received intraventricular injections of Rp-cAMP or a vehicle 10–15 min prior to 3 consecutive days of stress induction. **b** The effect of Rp-cAMP infusion on the AMPA/NMDA ratio in OFC–BLA synapses in control and stressed mice. Vehicle-treated control animals (Ctrl-Veh): *n* = 12 cell from 3 mice, Rp-cAMP-treated control animals (Ctrl-Rp): *n* = 12 cell from 3 mice, vehicle-treated stressed animals (Stress-Veh): *n* = 12 cells from 4 mice, Rp-cAMP-treated stressed animals (Stress-Rp): *n* = 12 cells from 4 mice. **c** The effects of Rp-cAMP infusion on the rectification index of AMPAR-mediated current (EPSC at −60 mV/EPSC at +60 mV) in the OFC–BLA synapse in control and stressed mice. Ctrl-Veh: *n* = 14 from 4 mice, Ctrl-Rp: *n* = 13 from 4 mice, Stress-Veh: *n* = 12 cells from 4 mice, Stress-Rp: *n* = 12 cells from 4 mice. **d** Time schedule of experiments examining the effect of PKA inhibition in the BLA on stress-induced behavioural changes. Mice received intra-BLA injections of Rp-cAMP or a vehicle 10–15 min prior to stress induction for 3 consecutive days. **e**, **f** The effect of PKA inhibition in the BLA on immobility in TST (**e**) and FST (**f**) in control and stressed animals. Ctrl-Veh: *n* = 15, Veh-Rp: *n* = 14, Stress-Veh: *n* = 14, Stress-Rp: *n* = 15. *P < 0.05, **P < 0.01, Tukey’s HSD test.

A previous study suggested that stress or glucocorticoid-induced insertion of CP-AMPARs into the hippocampus is mediated by a PKA-dependent mechanism^20^. We examined whether pharmacological inhibition of PKA during stress would block synaptic changes in the OFC–BLA synapse and behavioural abnormalities. Mice were injected with the PKA inhibitor Rp-cAMP, or vehicle in the lateral ventricle 10–15 min prior to each stress session (Fig. 3a) and electrophysiology was performed after three consecutive stress-inducing sessions. Vehicle-treated stressed animals had a significantly greater AMPA/NMDA ratio (Fig. 3b, stress × drug interaction: *F*[1,44] = 6.057, *P* = 0.018, two-way ANOVA. *P* < 0.01, Tukey’s HSD test) and inward rectification (Fig. 3c, stress × drug interaction: *F*[1,47] = 4.506, *P* = 0.039, two-way ANOVA. *P* < 0.05, Tukey’s HSD test, Supplementary Fig. 3A, at +60 mV, *P* < 0.01, Tukey’s HSD test) compared with the other groups. These data indicate that Rp-cAMP treatment had no effects on the AMPA/NMDA ratio and inward-rectification in control animals and prevented stress-induced changes in these synaptic functions in stressed animals. Mice were injected with Rp-cAMP, or a vehicle in the BLA 10–15 min prior to each stress session (Fig. 3d and Supplementary Fig. 3B). TST and FST were performed after three consecutive stress-inducing sessions. Vehicle-treated stressed animals displayed significantly higher immobility in TST (Fig. 3e, stress × drug interaction: *F*[1,54] = 4.677, *P* = 0.035, two-way ANOVA. *P* < 0.01, Tukey’s HSD test) and FST (Fig. 3f, stress × drug interaction: *F*[1,54] = 5.183, *P* = 0.027, two-way ANOVA. *P* < 0.05, Tukey’s HSD test) compared with the other groups. These data indicate that Rp-cAMP treatment had no effects on immobility in control animals and prevented the stress-induced increment of immobility in stressed animals. These data suggest that a PKA-dependent mechanism is involved in the stress-induced postsynaptic changes in the OFC–BLA pathway and stress-induced behavioural abnormalities.

### Optogenetic activation of OFC–BLA transmission increases, and chemogenetic inactivation decreases, stress-related behaviour

**Fig. 4:**
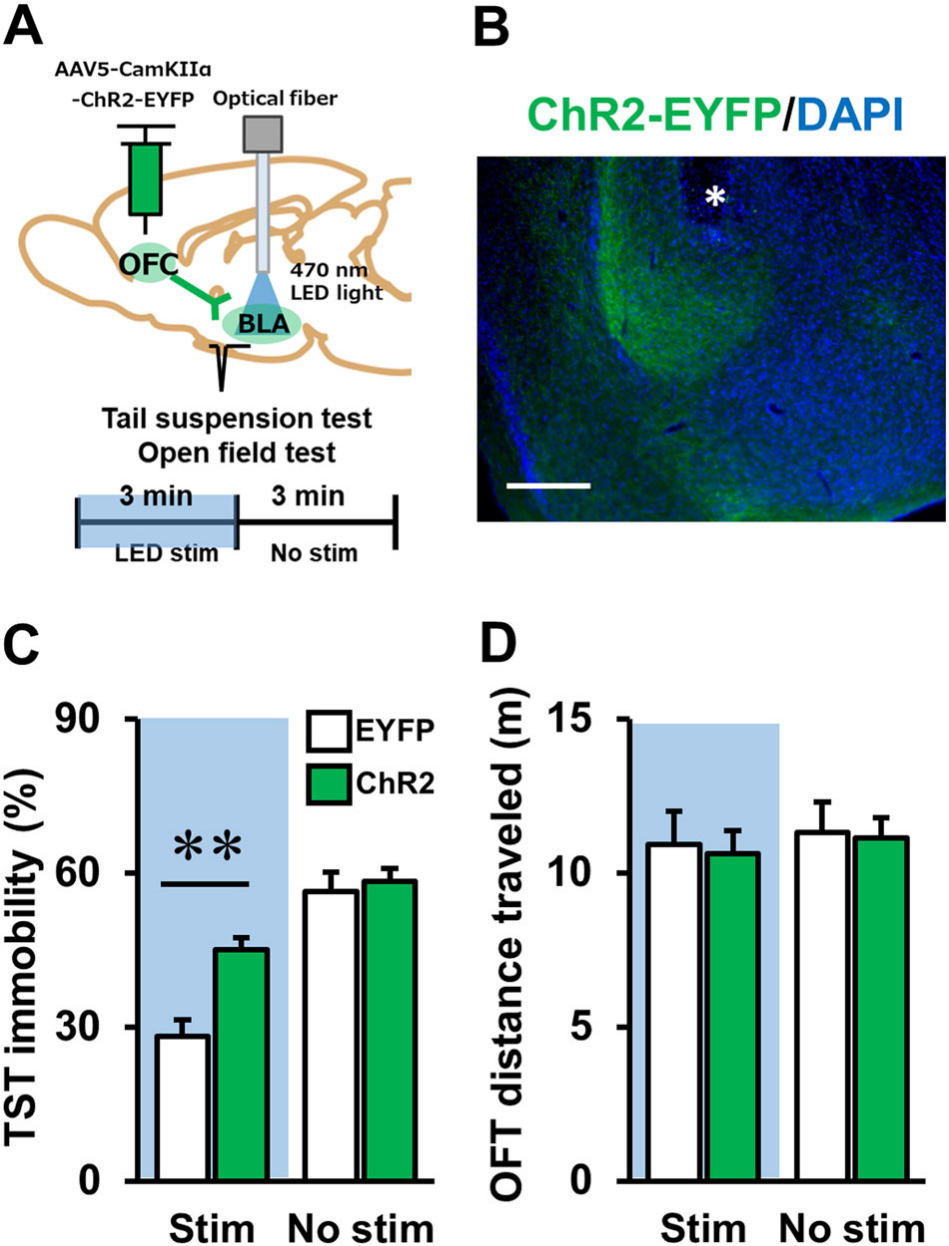
Optogenetic activation of the OFC–BLA pathway increased stress-related behaviour in stress-naive mice. **a** Schematic of viral injection and LED optic fibre placement for optogenetic activation of OFC–BLA transmission (top), and the experimental procedure to activate the OFC–BLA pathway during the behavioural experiments (bottom). TST and OFT were performed with optical stimulation in the first half (3 min) and without stimulation in the second half (3 min) of the test session. **b** Representative photograph of ChR2-EYFP expressing axon terminal from OFC and LED optic fibre tips in the BLA. Asterisk represents fibre tip. Scale bar: 250 μm. **c**, **d** The effect of activating the OFC–BLA pathway on immobility in the TST (**c**) and locomotor activity in the open field test (OFT, **d**). In the first half of the session, mice were optogenetically stimulated (Stim, blue shaded area), and in the last half of the session, optogenetic stimulation was not delivered to the mice (No Stim, no shaded area). EYFP: *n* = 10, ChR2: *n* = 13. **P < 0.01, unpaired *t*-test.

Pharmacological PKA inhibition during stress blocked synaptic change in the OFC–BLA pathway and reduced immobility in TST and FST in stressed mice. These findings prompted us to hypothesise that stress-induced change in the OFC–BLA synapse directly elicits stress-induced behavioural changes. To address the possible causal relationship between changes in OFC–BLA transmission and display of stress-related behaviour, we activated OFC–BLA transmission during TST using an optogenetic method in stress-naive mice. We expressed ChR2-EYFP in mice OFC using an AAV vector and implanted an optical fibre into the BLA for optogenetic stimulation (Fig. 4a, b and Supplementary Fig. 4A). After recovery from surgery, each mouse was subjected to TST with optogenetic stimulation (first half of the test session: Stim, Fig. 4a, c) or without stimulation (last half 3 min of the test session: No Stim, Fig. 4a, c) in the same test session. Notably, during the Stim period, ChR2 expressing mice displayed increased immobility in TST compared to EYFP expressing mice (Fig. 4c, *t*(23) = 4.425, *P* < 0.01, Student’s *t*-test). Contrastingly, no significant difference between groups was seen during the No Stim period (Fig. 4c, *t*(23) = 0.108, *P* = 0.915, Student’s *t*-test). As previous studies^43,44^ suggested that the duration of immobility progressively increases over time in TST, both EYFP and ChR2 expressing animals displayed increased immobility time in the first half of the session (Stim period) than in the last half (No Stim period). No significant difference was seen in locomotor activity in the OFT during the Stim or No Stim periods (Fig. 4d, Stim: *t*(23) = −0.612, *P* = 0.547, Student’s *t*-test, No Stim: *t*(23) = −0.906, *P* = 0.374, Student’s *t*-test).

**Fig. 5:**
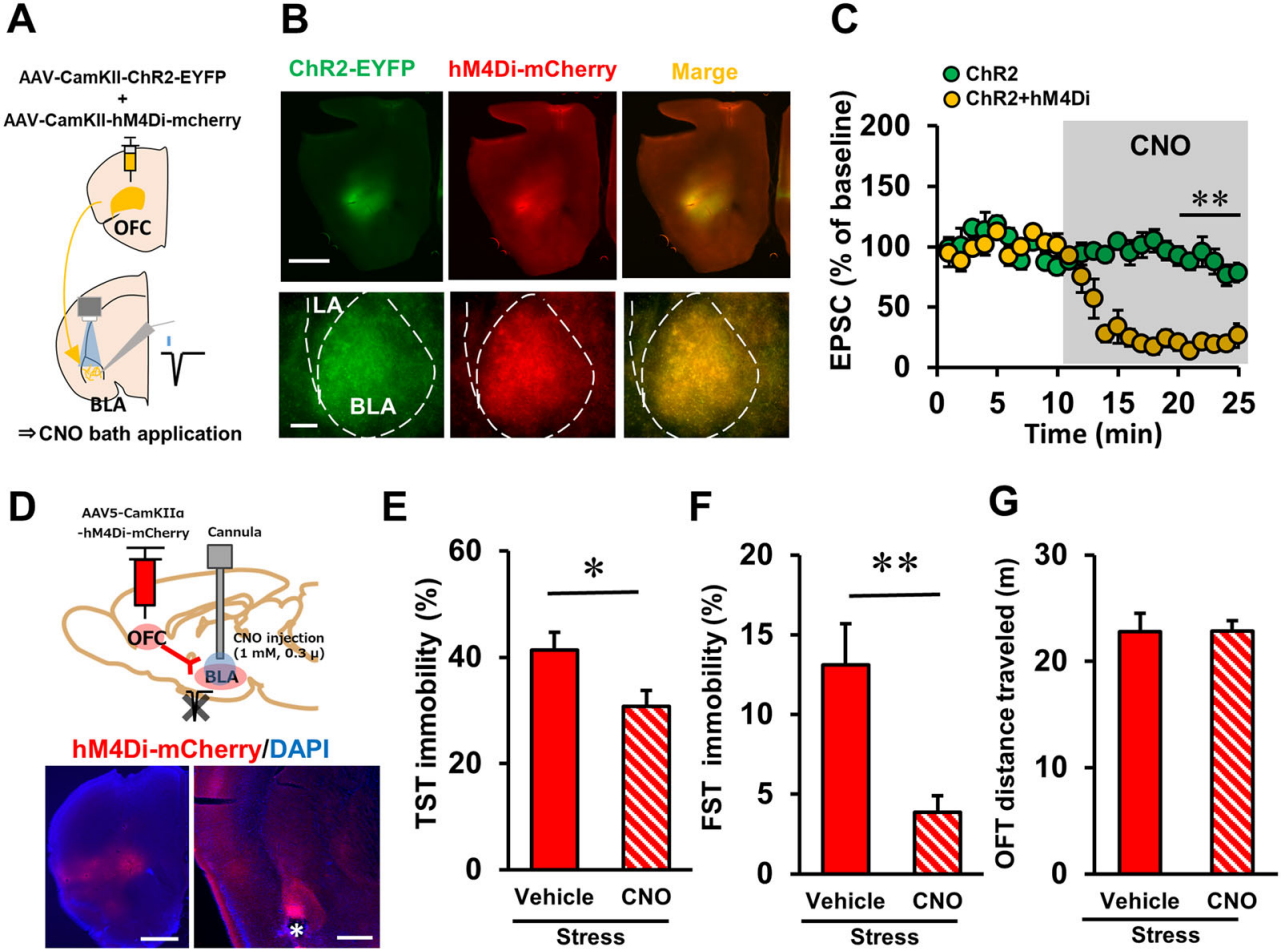
Chemogenetic inactivation of OFC–BLA transmission reduced stress-related behaviour in stressed mice. **a** A schematic of confirmation of the effect of CNO/hM4Di-induced axonal inactivation on the OFC–BLA synaptic response. **b** A representative photograph of ChR2-EYFP/hM4Di-mCherry fluorescence in the injection site in the OFC (top, scale bar: 1000 μm), and a ChR2-EYFP/hM4Di-mCherry co-expressing axon extending from the OFC into the BLA (bottom, scale bar: 250 μm). **c** The effect of CNO (50 μM) on the light-evoked OFC–BLA synaptic response in ChR2 expressing and ChR2/h4Di co-expressing mice. ChR2: *n* = 7 cells from 3 mice, ChR2/hM4Di: *n* = 6 cells from 3 mice. **d** Schematic of viral injection and cannula placement for chemogenetic inactivation of OFC–BLA transmission (top), and representative photographs of hM4Di-mCherry fluorescence in the viral injection site in the OFC, and hM4Di-mCherry expressing afferent axon from the OFC and injection cannula tips in the BLA (bottom). Asterisk represents injection needle tip in the BLA. **e**–**g** The effect of chemogenetic inactivation of the OFC–BLA pathway on immobility in the TST (**e**, vehicle: *n* = 13, CNO: *n* = 10), FST (**f**, vehicle: *n* = 13, CNO: *n* = 10), and locomotor activity in the OFT (**g**, vehicle: *n* = 11, CNO: *n* = 10) in stressed mice. *P < 0.05, **P < 0.01, unpaired *t*-test.

To examine the effects of inactivating OFC–BLA transmission on stress-related behaviour, we performed chemogenetic inactivation of this pathway in stressed mice. To confirm chemogenetic axonal inhibition in BLA, we injected a mixture of AAV vectors encoding ChR2-EYFP and hM4Di-mCherry into mice OFC (Fig. 5a). Four to six weeks after injection, ChR2-EYFP/hM4Di-mCherry co-expressing axon terminal was observed in the BLA (Fig. 5b). We conducted whole-cell recordings in BLA pyramidal neurons and examined the suppressive effect of CNO bath application on the OFC–BLA synaptic response (Fig. 5a). Synaptic responses in ChR2/hM4Di co-expressing mice robustly decreased after 10 min bath application compared with ChR2 expressing mice (Fig. 5c, 21–25 min, *P* < 0.001, the main effect of AAV, two-way ANOVA, *P* < 0.01, Student’s *t*-test). To investigate the effects of the inactivation of OFC–BLA transmission on mouse behaviour, we expressed hM4Di-mCherry in mouse OFC using an AAV vector and implanted a guide cannula in the BLA for CNO microinjection (Fig. 5d and Supplementary Fig. 5). After recovery from surgery, mice received repeated tail-shock stress-induction procedures followed by TST and FST with pre-injection of CNO or vehicle into the BLA. CNO injection significantly decreased immobility in TST (Fig. 5e, *t*(21) = −2.287, *P* = 0.033, Student’s *t*-test) and FST (Fig. 5f, *t*(15.431) = −3.309, *P* = 0.005, Welch’s *t*-test) in CNO-treated mice compared with vehicle-treated mice. By contrast, there was no significant difference in locomotor activity in the OFT (Fig. 5g, *t*(19) = 0.922, *P* = 0.368, Student’s *t*-test). These results suggest that OFC–BLA transmission could modulate stress-related behaviours.

## Discussion

The OFC–amygdala pathway has been highlighted as a crucial circuit for stress-related emotional changes. However, the effects of stress on OFC–amygdala transmission has not previously been addressed. In the present study, we optogenetically isolated OFC–BLA excitatory synaptic transmission, and demonstrated an increase in the AMPA/NMDA ratio, and insertion of CP-AMPARs as stress-induced changes in synaptic transmission. Pharmacological blockade of PKA during stress was shown to prevent both stress-induced changes in the OFC–BLA synapse and stress-related behaviour. Optogenetic activation of OFC–BLA synaptic transmission increased, and chemogenetic inactivation decreased stress-related behaviour. These data suggest that OFC–BLA synaptic transmission could modulate emotional behaviours, and repetitive stress might alter behaviours through postsynaptic modifications in the OFC–BLA pathway. Our findings promote a novel interpretation of emotion-related neural circuitry and identify molecular mechanisms underlying the stress-induced behavioural change.

### Stress-induced synaptic change in the OFC–BLA transmission and emotional behaviours

In the present study, we identified stress-induced post-synaptic changes in OFC–BLA synapses. Our findings increase knowledge of a pivotal issue regarding how stress affects emotional neural circuitry. This is the first study to examine the effects of stress on OFC–BLA synaptic transmission, to our knowledge. In addition, we found that stress-induced an increased AMPA/NMDA ratio and recruitment of CP-AMPARs into the OFC–BLA synapse. Similar plasticity is observed in thalamo-amygdala or mPFC-amygdala synapses after fear conditioning^40,45,46^, or synapses in VTA and NAc after cocaine exposure^47^ and artificial induction of long term potentiation^48^. An increased AMPA/NMDA ratio would result in enhanced synaptic transmission mediated by AMPARs^49^, which means that stress increases the efficiency of synaptic transmission in OFC–BLA synapses. This increased efficiency might cause stress-related behavioural changes. Alternatively, the increased AMPA/NMDA ratio could result in reduced synaptic transmission mediated by NMDARs, which means that stress attenuates synaptic plasticity mediated by NMDARs in OFC–BLA synapses. This attenuation might cause stress-related behavioural changes. In terms of recruitment of CP-AMPARs into OFC–BLA synapses, however, the former possibility is more plausible based on the present study.

Previous studies have shown that the OFC is implicated in processing negative emotions in humans^1,9^, and stress-related behaviours in rodents^4,5,50,51^. However, the underlying neural circuitry has not been elucidated. In the present study, we manipulated transmission within the OFC–BLA pathway, and evaluated its effects on behaviour in mice, using optogenetic and chemogenetic approaches. We found that the OFC can modulate stress-related behaviours in mice through the pathway projecting to BLA. Collectively, the alterations in synaptic activity in the OFC–BLA pathway might be one of the neural bases for stress-induced behavioural alterations.

Recently, the lateral region of the OFC was theorised to be involved in sustaining negative thoughts and emotions in depressive patients, because neurons in this area responded to non-reward or unpleasant stimuli^1,52^. It is speculative, but these orbitofrontal non-reward neurons may modulate negative emotion through a pathway projecting to the amygdala. This possibility is supported by evidence that neurons in the OFC and BLA are important for the anticipation of aversive outcomes, and the lateral OFC is necessary for encoding information about expected aversive outcomes in the BLA^53,54^.

Recently, growing evidence has indicated that stress induces alterations in brain regions involving reward systems, such as the NAc and VTA, and contributes to stress-induced behavioural changes^55–57^. Several physiological and optogenetic studies have demonstrated that the BLA can modulate stress-related behaviours through these reward circuits^58,59^. The OFC may regulate stress-related behaviour through the neural population in the BLA that projects to these reward circuits. Future studies using retrograde and trans-synaptic techniques will address these possibilities.

### Contribution of PKA signalling, and insertion of CP-AMPARs into the BLA

PKA is the best-known key regulator of AMPAR trafficking^60^. Several studies have shown stress-induced changes in phosphorylation of PKA and AMPAR subunits in the amygdala^18,21,61^. Thus, PKA-dependent synaptic modifications in the amygdala may be implicated in stress-related behaviours. In the present study, pharmacological blockade of PKA during stress prevented stress-induced changes in the OFC–BLA synapse and behaviours. Correspondingly, a recent study demonstrated that local knockdown of A-kinase anchoring protein 150, the regulator of PKA, in the amygdala, prevented stress-induced synaptic and behavioural changes in mice^61^. These results suggest an enhancement in PKA-dependent AMPAR trafficking in the OFC–BLA synapse, rather than a decrease in synaptic NMDAR, as a molecular mechanism contributing to the increased AMPA/NMDA ratio.

CP-AMPARs, which lack edited-GluA2 subunits, have a greater calcium permeability and contribute to synaptic plasticity^60^. In the present study, we found PKA-dependent stress-induced recruitment of CP-AMPARs in the OFC–BLA synapse. A similar function of CP-AMPARs is demonstrated in hippocampal synapses^20^. In addition, chronic stress increases CP-AMPARs as shown by GluA1/GluA2 ratio in the amygdala and the local blockade of CP-AMPARs in the amygdala attenuates stress-induced behavioural changes in rodents^21^. This suggests that stress-induced recruitment of CP-AMPARs in the amygdala, including the OFC–BLA synapse, contribute to stress-related behavioural changes. CP-AMPARs enhanced synaptic Ca^2+^ influx in addition to NMDARs and contribute to NMDAR-dependent or independent forms of synaptic potentiation^20,60,62^. CP-AMPAR-gated Ca^2+^ influx also facilitates synaptic structural plasticity^63^. This suggests that stress induces behavioural alterations through CP-AMPAR-induced plasticity in the OFC–BLA synapse. Furthermore, CP-AMPARs allow calcium permeation at resting membrane potentials. These channels may serve as synaptic “tags”, supporting the consolidation of synapse-specific potentiation^46^. In several stress-related psychiatric disorders, a sustained negative mood is observed in patients. Interestingly, OFC and amygdala activities correlate with rumination, repetitive thinking focused on negative mood, in patients with stress-related disorders^64,65^. Synaptic tagging in the OFC–BLA pathway might play an important role in sustaining negative emotions.

Previous studies have reported that synaptic recruitment of CP-AMPARs to the amygdala, NAc, and hippocampus has been observed in stressed animals^20,21,66^. Interestingly, blockade or potentiation of the CP-AMPARs in NAc increased and decreased depression-like behaviours in chronic neuropathic pain rats, respectively^66^. These results indicate that CP-AMPARs in NAc have an attenuating effect on stress-related behaviours, in contrast to the amygdala^21^, and CP-AMPARs modulate stress-related behaviours in a region-specific manner.

### Stress-induced synaptic changes in the ACC–BLA pathway

The amygdala receives input from divergent brain regions involved in emotion, such as the PFC, thalamus, hippocampus, raphe, and VTA^67^. Stress may induce distinct plasticity in these individual pathways to the amygdala, and their cooperation may modulate stress-induced behavioural alterations. In the present study, we focused on afferent pathways from two distinct subregions in the PFC (the OFC and ACC) to the BLA and examined the effect of stress on synaptic transmission in these pathways. By contrast with the OFC–BLA synapse, we found that repeated tail-shock stress decreased the AMPA/NMDA ratio in the ACC–BLA synapse. These results indicate that stress-induced pathway-specific synaptic changes in two distinct PFC–amygdala pathways. Interestingly, in the case of BLA stimulation by electrodes, there was no significant difference in the AMPA/NMDA ratio between control and stressed animals. Local electrical stimulation would evoke all of these different inputs into BLA. Therefore pathway-specific synaptic changes might be masked. This pathway specificity in stress-induced synaptic modifications might be due to differences in function and responsiveness to stress in these PFC subregions. While OFC inactivation decreases immobility in the forced swim test in rats^5^, ACC lesion results in increased immobility in the forced swim test in mice^68^, suggesting that the OFC and ACC have opposing functions in relation to stress-related behaviours. Furthermore, a morphological study has suggested that stress increases dendritic arbour formation in OFC pyramidal neurons but decreases it in ACC^69^. These findings suggest that stress causes distinct neural changes in the OFC and ACC, and it affects downstream circuits projecting to BLA. Alternatively, the pathway-specific synaptic change may depend on the difference in postsynaptic neurons in the BLA. BLA neurons have functional, electrophysiological, and morphological features that display heterogeneity^70–72^, and a previous study has demonstrated that reward or fear conditioning induces opposite synaptic changes in distinct neuronal populations in the BLA^73^. Besides, chronic stress induces a dendritic change in some, but not all subtypes of neurons in the BLA^74^. Therefore, OFC and ACC might project to separated neuronal subsets in the BLA which have distinct synaptic responsiveness to stress.

### Clinical implications of stress-induced alterations in the OFC–BLA pathway

In human imaging studies, increased functional connectivity between the OFC and amygdala has been observed in depressive patients^14–16^. In the present study, we showed that repeated stress could change excitatory transmissions in the OFC–amygdala synapse. These synaptic changes might underlie abnormal functional connectivity between the OFC and amygdala in patients with stress-related disorders and might lead to emotional dysregulations. These speculations suggest the possibility that manipulating the neuroplasticity within the OFC–amygdala circuit could provide effective therapy for stress-related psychiatric disorders.

## Supplementary information

Supplementary information

Supplementary figure 1

Supplementary figure 2

Supplementary figure 3

Supplementary figure 4

Supplementary figure 5
