# Supplementary material for: Stress induces insertion of calcium-permeable AMPA receptors in the OFC–BLA synapse and modulates emotional behaviours in mice
Source: Transl Psychiatry. 2020 May 18;10:154. doi: 10.1038/s41398-020-0837-3 (PMC7235080; doi:10.1038/s41398-020-0837-3)
Supplement: Supplementary file 1 — Supplementary information [file 41398_2020_837_MOESM1_ESM.doc]

**Supplemental Information**

**Detailed materials and methods**

**Animals and stress procedure**

Male C57BL/6J mice (3-5 weeks of age at viral injection surgery, 8-12 weeks of age for electrophysiological experiment and behavioural tests, CLEA, Tokyo, Japan) were used. Animals were housed in groups of 3-4 in plastic cages under controlled temperature (23 ±1°C) and lighting (12h light/dark cycle, light onset at 08:00 AM) conditions with food and water ad libitum. Our experimental procedures complied with the guidelines of the National Institute of Neuroscience, and the National Center of Neurology and Psychiatry, and were approved by the Institutional Animal Investigation Committee.

Mice received repeated tail-shock stress (learned helplessness stress^24^) for 3 consecutive days. The animals were restrained in a plexiglas tube for 2h, and received 40 electric tail shocks (1 mA, 3 s duration) at varying intervals (140-180 s). Control mice were left in their home cages. All stress procedures were conducted during the light phase of the illumination cycle. For electrophysiological experiments, mice received repeated tail-shock stress four to seven weeks after viral injection. Mice were subjected to behavioural or electrophysiological testing within 2 days after their last stress session.

**Behavioural Tests**

All behavioural tests were conducted during the light phase of the illumination cycle. In all behavioural experiments, the animals in the same cage were randomly divided into the control and experimental groups. The animals in the experimental and control groups were tested alternately to avoid a diurnal variation bias. This ensured that both groups were tested in the same period of time. On the day of the test, mice were transported to the testing room and left in their home cages for at least 1 h before the test. To evaluate depression-like behaviour, we conducted forced swim and tail suspension tests. In the forced swim test, mice were subjected to a 6 min swimming session. During the session, mice were placed inside a 25-cm glass beaker (14 cm diameter) containing 20 cm of water maintained at 24±2°C, and the duration of their immobility was measured. A mouse was judged to be immobile if it floated motionless in the water making only the necessary movements to keep its head above water^75^. Following a swim session, mice were removed from the beaker, dried with paper towels, and returned to their home cages. The tail suspension test was performed for a duration of 6 min. During a session, a mouse was suspended by its tail with adhesive tape to an aluminium bar. The duration of its immobility was measured. Clear hollow cylinders cut from polycarbonate tubing were placed around the tails of the mice to prevent tail climbing behaviour. Mice were judged to be immobile if they remained motionless except for whisker movement and respiration^76^. In some experiments, the duration of immobility was manually scored by an investigator who did not know the animal group condition (Fig. 2C, D). In other experiments, the duration of immobility was scored by an investigator who knew the animal group condition (Fig, 3E, F, Fig. 4C D, Fig. 5E-G). To evaluate locomotor activity, we conducted open field tests. This procedure involved a 6 min session (Optogenetic experiment) or a 5 min session (Chemogenetic experiment). During the session, mice were left in an open-field arena (50 cm × 50 cm × 40 cm, O'Hara & Co., Ltd., Tokyo, Japan), and their distance travelled was quantitated using a software-driven automated system (TimeOFCR1, O'Hara & Co., Ltd). The light intensity was 200 lx at the centre of the arena. The apparatus was cleaned with 70% ethanol before each test.

**Stereotaxic surgery**

Animals were anaesthetised by intraperitoneal injection of ketamine (100 mg/kg) and xylazine (20 mg/kg) and positioned using a stereotaxic instrument (Narishige, Tokyo, Japan). Eyes were protected against drying by applying ointment (Tarivid ophthalmic ointment, Santen, Osaka, Japan). A small segment of the skull above the OFC or ACC (OFC: 2.6 mm anterior to bregma, and 1.6 mm lateral to the midline, ACC: 1.3 mm anterior to bregma, and 0.3 mm lateral to the midline) was removed. Virus solutions (AAV5-CaMKIIa-ChR2-EYFP, AAV5-CaMKIIa- EYFP purchased from the University of North Carolina Vector Core and AAV5-CaMKIIa-hM4Di-mCherry purchased from Addgene) were pressure-injected into the OFC or ACC (OFC: -1.8 mm from the dura, ACC: -0.9 mm from the dura, volume: 300 nl, infusion rate: 100 nl/ min) using a 10 μL Hamilton syringe driven by an infusion pump (UMP-3, World precision instruments, FL, USA). After injection, the needle was left in place for 10 min and then slowly retracted. The exposed cortex was covered with a gelatine sponge (Spongel, Astellas Pharma Inc., Tokyo, Japan), and the scalp was sutured closed.

For drug microinjections, guide cannulas were implanted (Eicom, Kyoto, Japan) into the lateral ventricle (0.05 mm posterior to bregma and 1.0 mm lateral to the midline, -1.5 mm from the dura, unilaterally) or the BLA (1.26 mm posterior to bregma, and 3.2 mm lateral to the midline, -3.5 mm from the dura, bilaterally), and attached to the skull with dental cement. For optogenetic activation, a dual-LED optic cannula (TeleLCD-B-5-500-6.2, BRC Nihon Bioresearch, Hashima, Japan) was implanted into the BLA (1.26 mm posterior to bregma, and 3.2 mm lateral to the midline, -3.5 mm from the dura, bilaterally), and attached to the skull with dental cement 3 to 4 weeks after AAV injection. After surgery, mice were housed individually and given 5–7 days to recover prior to stress induction or behavioural testing. All coordinates are based on the atlas of Paxinos and Watson (1998). After the behavioural tests, the locations of the injection or LED cannula were confirmed. If the locations of injection cannula were out of the BLA, these animals were excluded.

**Electrophysiology**

Whole-cell patch-clamp recording from pyramidal neurons in the amygdala was performed as previously described^25-28^. All electrophysiological experiments were performed without blinding. Brain slices (300 μm thick) containing the amygdala were prepared in artificial cerebrospinal fluid (aCSF; containing [in mM] 125 NaCl, 4.4 KCl, 1.5 MgSO4, 1.0 NaH2PO4, 26 NaHCO3, 10 glucose, 2.5 CaCl2), pH 7.4, 290–300 mOsm/L) using a linear slicer (Dosaka EM Co., Ltd., Kyoto, Japan). Slices were prepared at a 35° tilt from horizontal^41^. Before transfer to a recording chamber, slices were maintained for at least 30 min at room temperature in ACSF. Brain slices were perfused (3.0 ml/min, gravity flow) with ACSF maintained at 28–32°C with an in-line heater and an automatic temperature controller (Warner Instruments, Hamden, CT, USA). Patch-clamp recordings were taken from pyramidal-shaped principal neurons, which were surrounded by ChR2-EYFP positive fibres in the BLA. To activate ChR2, blue light (465 nm) was delivered to the recorded cell through a 63x objective lens (duration = 0.1 -3 ms, 1.8-70.7 x 10-7 J under the objective lens), using an LED lamp and its driver (LEX-2B, BrainVision, Tokyo, Japan). Synaptic responses obtained by electrical stimulation were elicited with a stimulating bipolar electrode (CBABD50, FHC, Bowdoin, ME, USA), which was placed within the BLA. Patch electrodes (resistance 4–7 MΩ) were filled with a solution containing the following: (in mM) 132 K-gluconate, 3 KCl, 10 HEPES, 0.5 EGTA, 1 MgCl2, 12 Na- phosphocreatine, 3 Mg-ATP, 0.5 Na-GTP, pH 7.4 with KOH, 285–290 mOsM or 105 CsOH, 30 CsCl, 10 HEPES, 0.5 EGTA, 1 MgCl2, 12 Na-phosphocreatine, 3 Mg-ATP, 0.5 Na-GTP, pH 7.3 with gluconic acid, 295 mosM. The electrophysiological signal was amplified and filtered at 5 kHz using a MultiClamp 700B patch-clamp amplifier (Axon Instruments, Union City, CA, USA). Data were digitised at 50 kHz and acquired using Clampex software (version10.6, Axon Instruments). The access resistance, which was frequently checked during recording, was between 10 and 25 MΩ. Cells with large drifts (±20%) in resistance were excluded from the analysis. To check whether the light-evoked excitatory postsynaptic current (EPSC) from the OFC to the BLA was ChR2-dependent, we blocked the voltage-gated sodium channels with 1 μM tetrodotoxin (TTX), then applied an additional 1 mM 4-aminopyridine (4-AP) to block the K channels that are critical for axon repolarisation^33^. To record the AMPA/NMDA ratio, picrotoxin (100 μM)) was included in the aCSF. The amplitude of the AMPAR-mediated EPSC was measured at -70 mV. The amplitude of the NMDAR-mediated EPSC was measured at +40 mV, 60 ms after exposure to blue light. For measurement of AMPAR rectification, picrotoxin (100 μM)) and AP-5 (50 μM) were included in the aCSF and 100 μm spermine was added in the internal solution. The peak amplitude for AMPAR-mediated EPSC was reported as a percentage of the value at −80 mV for analysis of AMPAR-rectification. The rectification index was calculated as the ratio of peak current amplitude at -60 mV and +60 mV. To detect the presence of calcium-permeable AMPA receptors (CP-AMPARs), a selective blocker of CP-AMPARs, NASPM (50 μM) was perfused. To confirm the effect of chemogenetic axonal inhibition on the OFC-BLA synaptic response, clozapine n-oxide (CNO, 50 µM) was perfused.

**Drug microinjection**

Before drug infusion, mice were gently restrained, and dummy cannulas were replaced with injection cannulas that extended 1 mm from the tip of the guide cannula. To inhibit PKA during stress induction, saline or Rp-cAMP (37 nmol /μL in saline, volume; 3 µL, infusion rate: 1 μL/min for intraventricular infusion, or volume: 0.3 μL, infusion rate: 0.1 µL/min for intra-BLA infusion^77^) were infused using a 10 μL Hamilton syringe controlled by an infusion pump (UMP-3, world precision instruments). For chemogenetic inhibition of OFC- BLA transmission, saline or clozapine n-oxide (CNO, 1 mM, volume: 0.3 μL^32^) was infused into the BLA. The injection needle remained in place for one additional minute after infusion to allow for proper diffusion. The mice received drug infusions 10-15 min before stress induction or behavioural testing.

**Optogenetic stimulation**

Optogenetic stimulation was performed using a wireless optogenetic stimulation system (Teleopto, BRC Nihon Bioresearch). Before the behavioural tests, an infrared receiver (TeleR-2-P, BRC Nihon Bioresearch) was connected to the LED cannula implanted in the virus-infected mice. Blue light stimulation (470 nm, 8-14 mW, 10ms, 10Hz) was delivered under the control of an LED remote controller (TeleRemocon, BRC Nihon Bioresearch) and an electronic stimulator (SEN-7203, Nihon Koden, Tokyo, Japan) during test sessions. TST and OFT were performed with optical stimulation in the first half (3min) of the test session and without stimulation in the second half (3 min).

**Supplemental figures**

**Figure S1, ChR2 expressing ACC-BLA projection axon and effects of repetitive tail-shock on the AMPA/NMDA ratio obtained by intra-BLA electrical stimulation in BLA pyramidal neurons**

(A) Representative photograph of ChR2-EYFP fluorescence at the injection site at ACC (left, scale bar: 1000 μm) and a ChR2-EYFP expressing ACC axon in the BLA (right, scale bar: 250 μm). (B) Schematics of intra-BLA electrical stimulation. (C) Representative traces of AMPAR and AMPAR/NMDAR mixed EPSCs obtained by intra-BLA electrical stimulation. (D) The effects of repetitive tail-shock on AMPA/NMDA ratios obtained by intra-BLA electrical stimulation. Ctrl: n = 11 cells, Stress: n = 11 cells. There was no significant difference between these two groups (Mann-Whitney U-test). Scale: 20 ms and 100 pA.

**Figure S2, The effect of repetitive tail-shock on the paired-pulse ratio in OFC-BLA and ACC-BLA synapses.**

(A) Representative traces of EPSCs evoked by paired optic stimulation with a 50 ms interpulse interval in the OFC-BLA synapse. (B) Effects of repetitive tail-shock on the paired-pulse ratio in the OFC-BLA synapse at various interpulse intervals. Ctrl: n = 14 cells, Stress: n = 16 cells. There was no significant difference between the two groups (Student’s t-test). (C) Representative traces of EPSCs evoked by paired optic stimulation with a 50 ms interpulse interval in the ACC-BLA synapse. Scale: 10 ms and 100 pA. (D) Effects of repetitive tail-shock on the paired-pulse ratio in the ACC-BLA synapse at various interpulse intervals. Ctrl: n = 12 cells, Stress: n = 10 cells. There was no significant difference between the two groups (Student’s t-test). Scale: 20 ms and 100 pA.

**Figure S3, The effect of Rp-cAMP infusion on the current-voltage relationship in the OFC-BLA synapse and injection needle tip locations in Rp-cAMP and vehicle-treated mice, relative to Figure 3.**

(A) The effects of Rp-cAMP on the current-voltage relationship of AMPAR-mediated current in the OFC-BLA synapse in control and stressed mice. Ctrl-Veh: n = 14 from 4 mice, Ctrl-Rp: n = 13 from 4 mice, Stress-Veh: n = 12 cells from 4 mice, Stress-Rp: n = 12 cells from 4 mice. **: P < 0.01, Stress-Veh vs other all groups, ##: P < 0.01, Ctrl-Veh vs Stress-Veh, Tukey’s HSD test. (B) Schematic of injection needle tip locations in BLA in Rp-cAMP and vehicle-treated control (left) and stressed (right) mice.

**Figure S4, Schematic of OFC viral injection sites, and optic fibre placements for optogenetic stimulation relative to Figure 4.**

Locations of viral injection sites in the OFC, and optic cannula placements in the BLA for mice in Fig. 4. Grey and green circles represent the EYFP and ChR2- EYFP expressing mice, respectively.

**Figure S5, Schematic of OFC viral injection sites and injection cannula placements relative to Figure 5.**

Locations of viral injection sites in the OFC, and injection cannula tip placements in the BLA of mice in Fig. 5. Grey and red circles represent the vehicle and CNO-treated mice, respectively.

**Supplemental References**

75. Petit-Demouliere, B., Chenu, F. & Bourin, M. Forced swimming test in mice: a review of antidepressant activity. *Psychopharmacology* **177**, 245-255 (2005).

76. Mueller, B. R. & Bale, T. L. Sex-specific programming of offspring emotionality after stress early in pregnancy. *J. Neurosci.* **28**, 9055-9065 (2008).

77. Bourtchouladze, R. et al. Different training procedures recruit either one or two critical periods for contextual memory consolidation, each of which requires protein synthesis and PKA. *Learn. Mem.* **5**, 365-374 (1998).
