# Supplementary figures and images for: Stress induces insertion of calcium-permeable AMPA receptors in the OFC–BLA synapse and modulates emotional behaviours in mice
Source: Transl Psychiatry. 2020 May 18;10:154. doi: 10.1038/s41398-020-0837-3 (PMC7235080; doi:10.1038/s41398-020-0837-3)

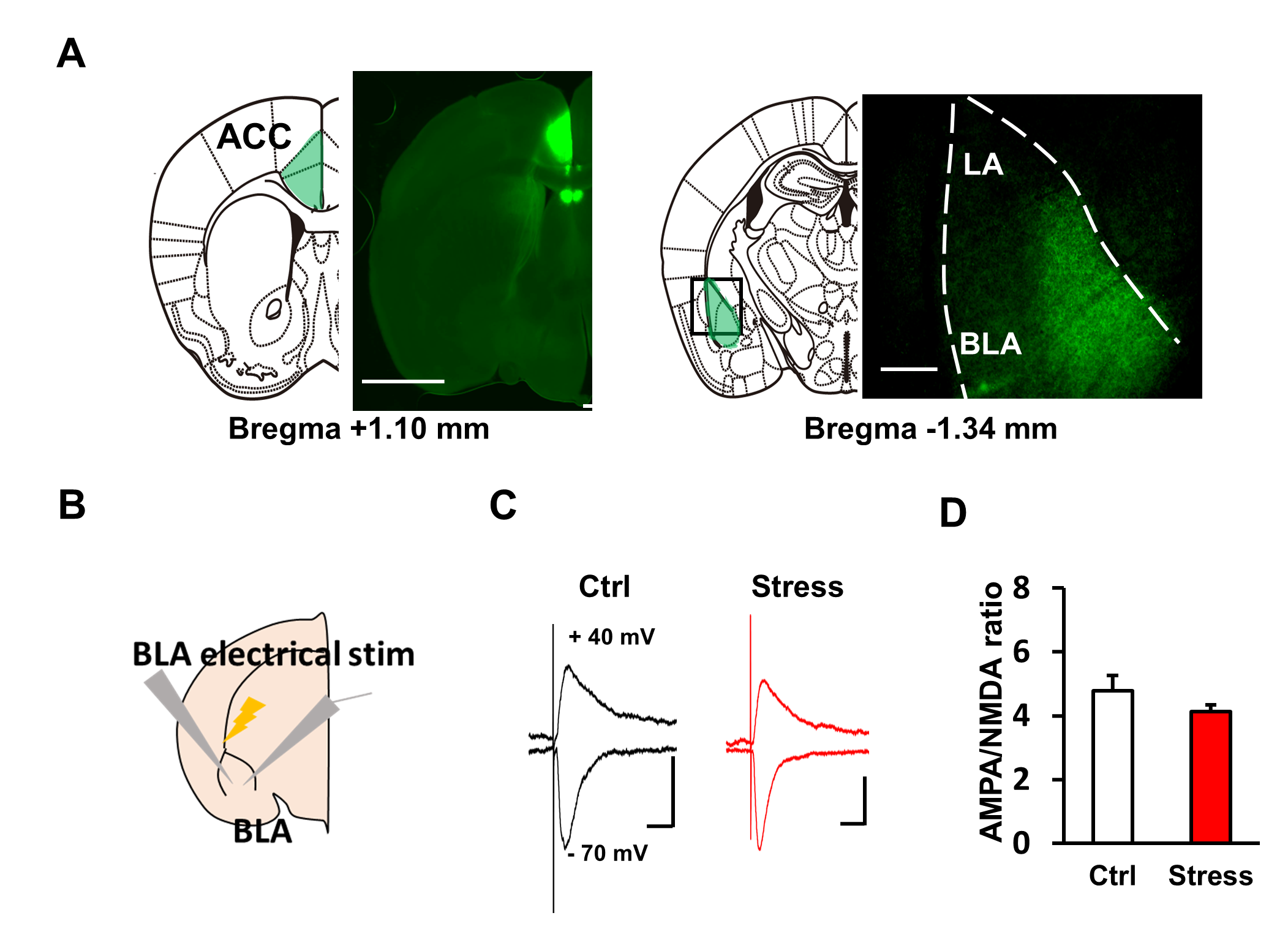

Supplement: Supplementary file 2 — Supplementary figure 1 [file 41398_2020_837_MOESM2_ESM.tif]

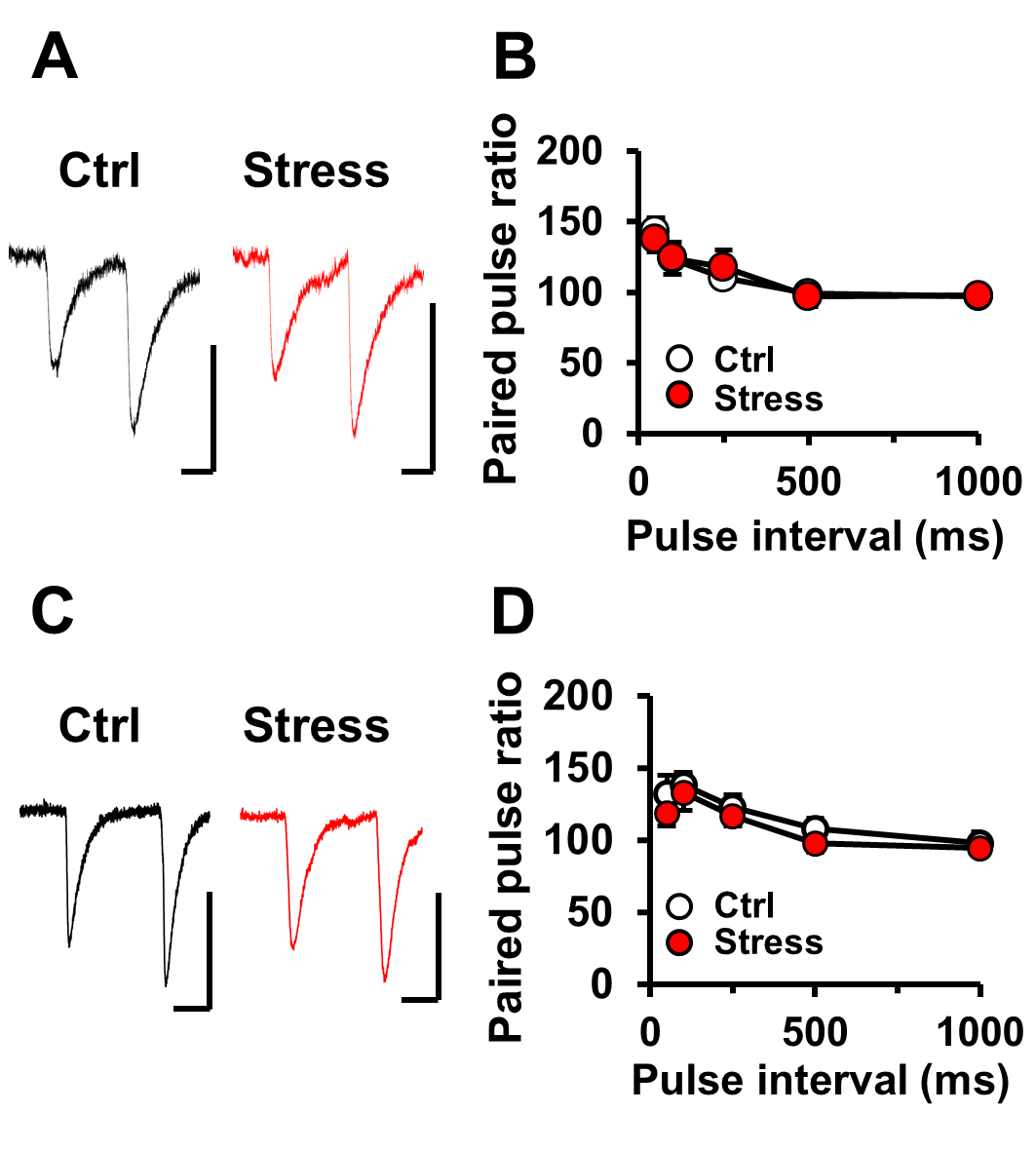

Supplement: Supplementary file 3 — Supplementary figure 2 [file 41398_2020_837_MOESM3_ESM.tif]

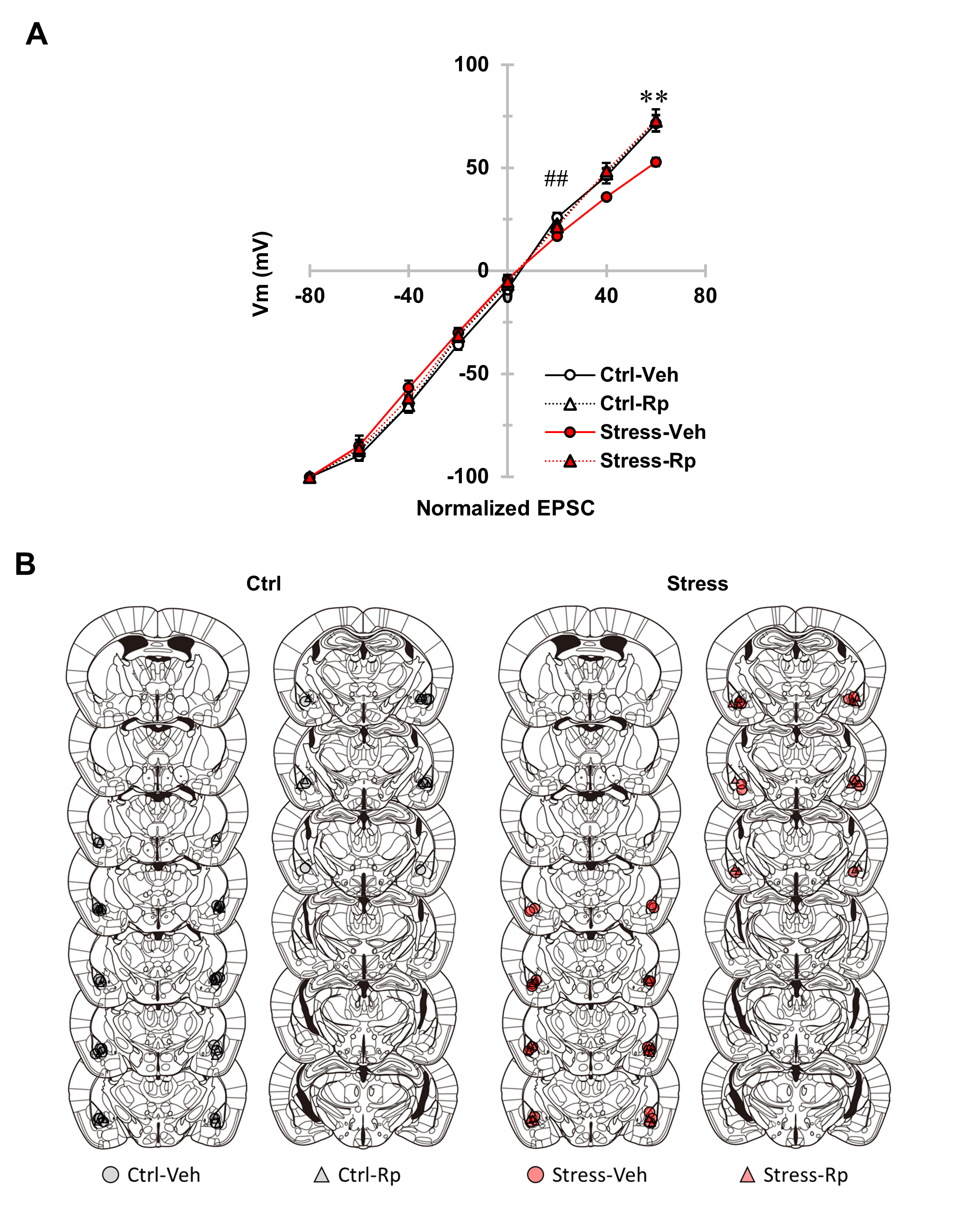

Supplement: Supplementary file 4 — Supplementary figure 3 [file 41398_2020_837_MOESM4_ESM.tif]

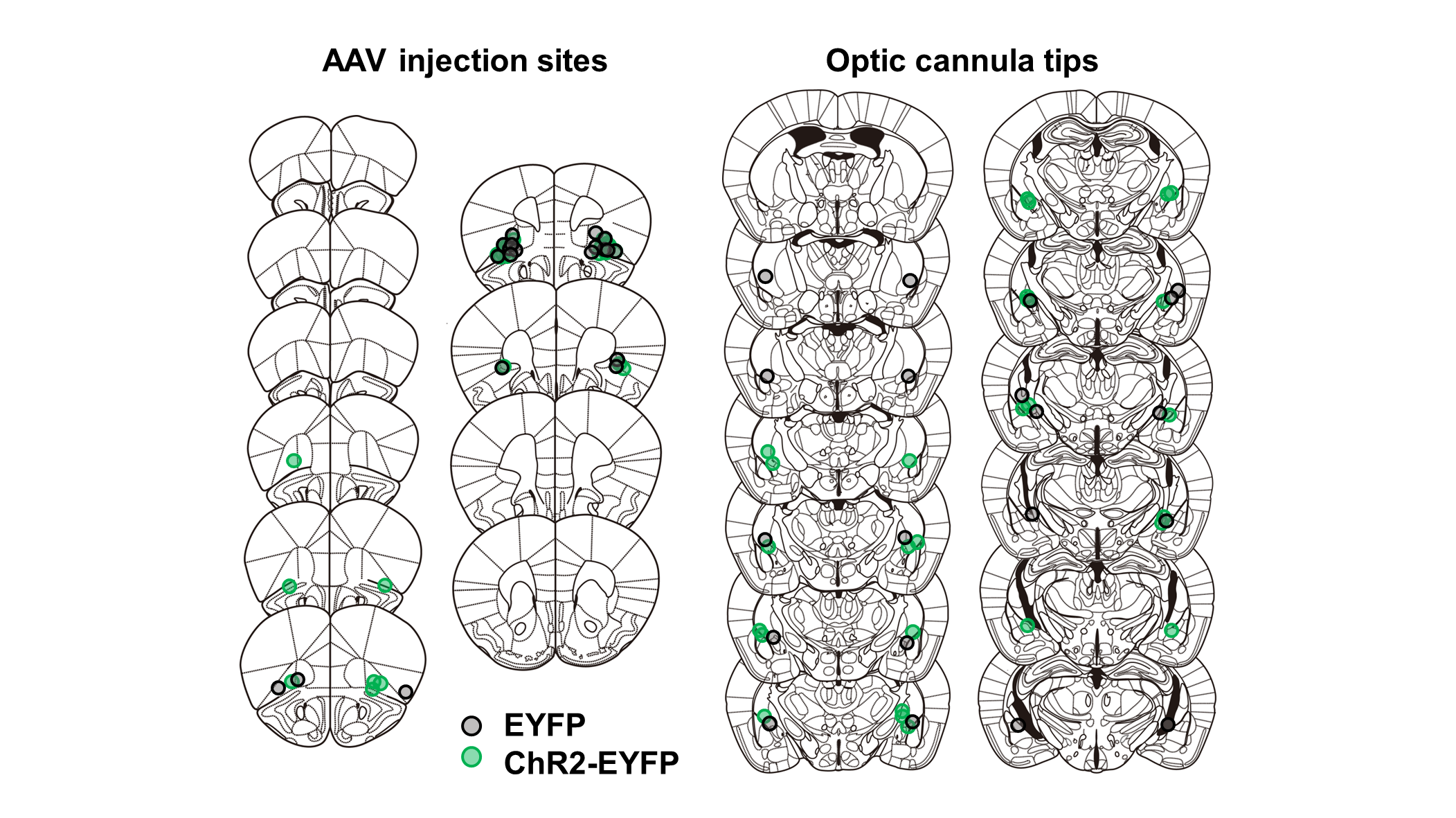

Supplement: Supplementary file 5 — Supplementary figure 4 [file 41398_2020_837_MOESM5_ESM.tif]

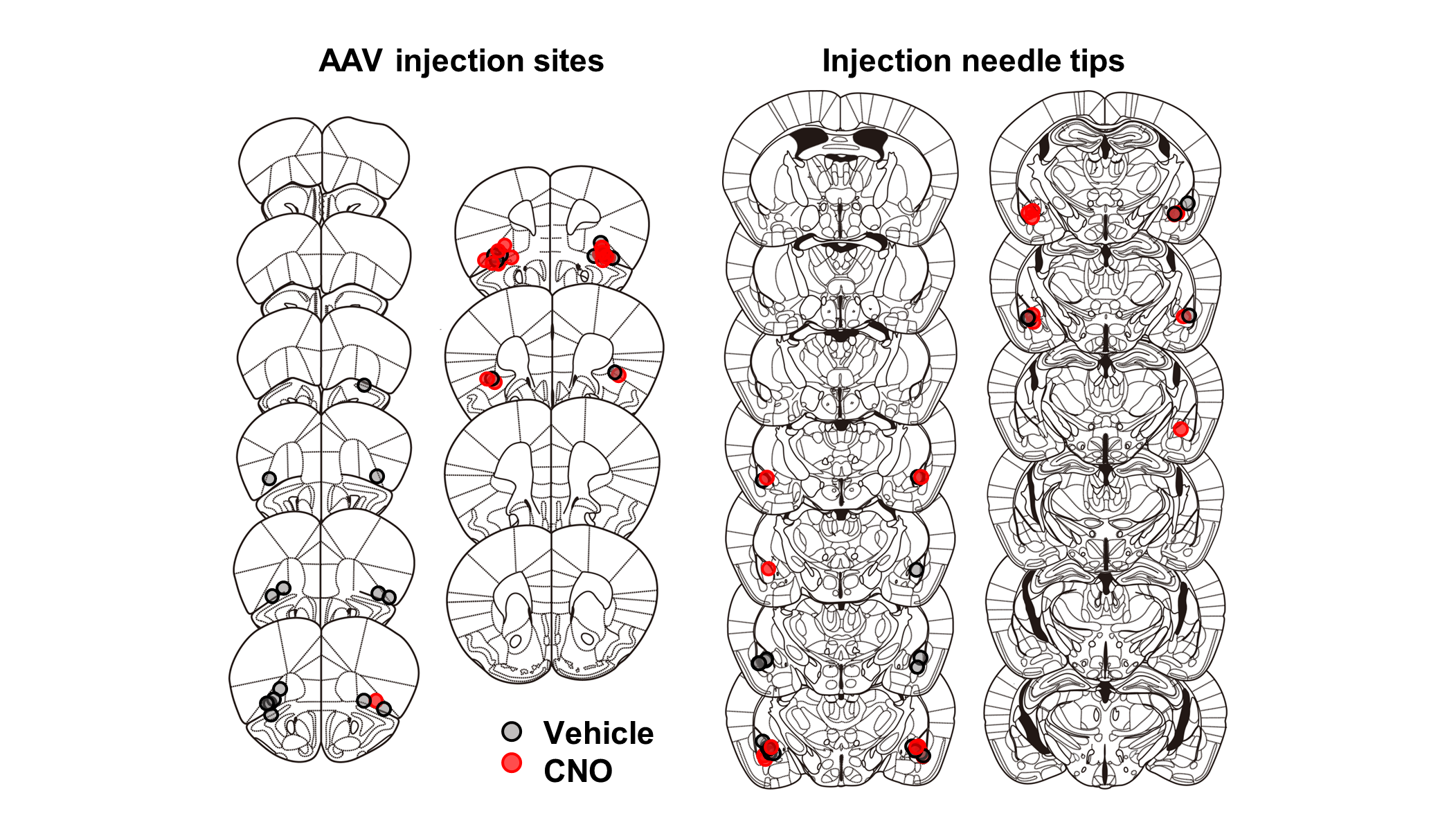

Supplement: Supplementary file 6 — Supplementary figure 5 [file 41398_2020_837_MOESM6_ESM.tif]
